# Structural analysis of ITS 1 gene of *Leishmania tropica* and evaluation of a novel ligand, benzo[d][1,3]dioxol-5-yl 4-acetamidobenzenesulfonate, via molecular modeling methods

**DOI:** 10.3389/fcimb.2026.1743630

**Published:** 2026-03-03

**Authors:** Mehmet Murat Yasar, Ekrem Yasar, Nuri Yorulmaz, Gulcan Gurses, Habip Celik, Murat Guney, Ahmet Tas, Zubeyde Tanriverdi, Nebiye Yentur Doni

**Affiliations:** 1 Vocational School of Health Services, Harran University, Sanliurfa, Türkiye; 2 Department of Biophysics, Faculty of Medicine, Erzincan Binali Yildirim University, Erzincan, Türkiye; 3 Physics Department, Faculty of Arts and Sciences, Harran University, Sanliurfa, Türkiye; 4 Department of Chemistry, Faculty of Science and Art, Agri Ibrahim Cecen University, Agri, Türkiye; 5 Faculty of Pharmacy, Agri Ibrahim Cecen University, Agri, Türkiye; 6 Department of Pharmaceutical Toxicology, Faculty of Pharmacy, Agri Ibrahim Cecen University, Agri, Türkiye; 7 Department of Clinical Microbiology, Faculty of Medicine, Harran University, Sanliurfa, Türkiye

**Keywords:** anti-leishmanial drug, leishmaniasis, *L. tropica*, molecular docking, molecular dynamics simulation, benzo[d][1,3]dioxol-5-yl 4-acetamidobenzenesulfonate

## Abstract

**Introduction:**

Leishmaniasis, a prevalent tropical disease caused by intracellular protozoa of the genus *Leishmania*, poses significant health challenges globally, exacerbated by migration waves from endemic regions. Despite its widespread impact, an effective vaccine for leishmaniasis remains elusive. Historically, antimony compounds have been employed in its treatment, but the emergence of resistant strains necessitates the development of new therapeutic agents. Addressing this need, our study focused on the structural characterization of a previously uncharacterized protein from *Leishmania tropica* using computational biomolecular techniques.

**Methods:**

We identified and docked the ligand benzo[d][1,3]dioxol-5-yl 4-acetamidobenzenesulfonate (3) and synthesized the reaction of sesamol (1) with sulfonyl chloride (2), and the NMR and IR spectra were used for characterization, a potential inhibitor of this protein, followed by a 300-ns simulation using the GROMACS software.

**Results:**

The results showed that the protein structure in the ITS1 gene region of *L. tropica* consisting of 875 amino acids was effectively inhibited. In addition, based on the broad pharmacological properties of sesamol and sulfonate esters, as well as the results obtained from ProTox-III analysis, compound 3 was synthesized and its effect on *L. tropica* was investigated. This evaluation was further supported by DataWarrior, SwissADME, and ADMETlab analyses. The ligand’s moderate binding affinity (Δ*G* = −6.29 kcal/mol), the formation of multiple hydrogen bonds (*n* = 4), its sustained binding throughout the 300-ns simulation, and the observed decrease in root mean square fluctuation (RMSF) values collectively support the idea that the synthesized compound may act as a potential inhibitor.

**Conclusion:**

However, experimental studies are required to conclusively confirm its inhibitory efficacy. This study provides valuable insights for the development of new therapeutic approaches against leishmaniasis.

## 1 Introduction

Leishmaniasis, a vector-borne disease caused by various species of *Leishmania*, is transmitted through the bite of infected female sandflies. The disease manifests in three primary forms: visceral leishmaniasis (VL), also known as kala-azar, which involves internal organs; cutaneous leishmaniasis (CL), the most common form that causes skin lesions; and mucocutaneous leishmaniasis with involvement of the mucous membranes of the upper respiratory and aerodigestive tracts including oral cavity, pharynx, and larynx. According to the World Health Organization (WHO), approximately 30,000 new cases of VL and over 1 million new cases of CL are reported annually. Approximately 95% of CL cases occur in the Americas, the Mediterranean basin, the Middle East, and Central Asia. Extensive research has been conducted on leishmaniasis, a disease targeted for global elimination by the WHO (WHO, 2024). In Türkiye, the majority of CL cases are concentrated in Şanlıurfa, Adana, Gaziantep, Hatay, Osmaniye, Kahramanmaraş, and Mersin. The influx of refugees from the Syrian conflict has led to an increase in the number of cases in southeastern provinces, exacerbated by irregular migration and challenges in early diagnosis and treatment (Gurses et al., 2018; Doni et al., 2020). In Türkiye, *Leishmania tropica* (*L. tropica*), *Leishmania major* (*L. major*), and *Leishmania infantum* (*L. infantum*) are the primary causative agents of CL (Gurses et al., 2018; Doni et al., 2020; Tunalı and Özbilgin, 2023). The promastigote form of *Leishmania*, characterized by a motile flagellum, develops in the sandfly vector. Upon transmission to the human host via a sandfly bite, the parasite transforms into the nonmotile amastigote form. The incubation period ranges from 2 to 8 weeks, beginning with an erythematous papule at the bite site, which enlarges into a nodule, eventually ulcerating and crusting over (Markle and Makhoul, 2004). While numerous therapeutic options have been explored in recent years, few have demonstrated consistent efficacy and safety (Minodier and Parola, 2007; Mokni, 2019). The susceptibility of *Leishmania* species can differ to available treatments of the disease (de Vries et al., 2015).

Conventional treatment for CL involves the administration of antimony compounds, such as meglumine antimoniate (Glucantime) and sodium stibogluconate (Pentostam), as well as amphotericin B, paromomycin, pentamidine, and oral miltefosine, either topically or parenterally (Sundar et al., 2001; Singh et al., 2016; Sundar and Singh, 2018; Passero et al., 2021; Doni and An, 2024). The use of standard antileishmanial drugs is restricted due to factors like high cost, toxicity, long treatment duration, treatment failure, and the emergence of antileishmanial drug resistance (Sundar et al., 2001; Singh et al., 2016; Sundar and Singh, 2018; Passero et al., 2021).

In anti-leishmanial drug research, some diselenides have been evaluated using quantitative structure–activity relationship (QSAR), pharmacokinetic analysis, density functional theory (DFT), molecular docking, and molecular dynamics simulation (MDS), demonstrating their potential as scaffolds for new drugs (Ugbe et al., 2024). MDS methods have been known to be invaluable in identifying potential ligands for different *Leishmania* species (Vijayakumar et al., 2024). In Ranjan and Dubey’s study, *in silico* techniques such as molecular docking, pharmacokinetics analysis, and MDSs were utilized to identify efficacy inhibitors including Staurosporine, Solasodine, Cromolyn, and Oxetacaine against the *Leishmania donovani* citrate synthase, which is relatively less explored but essential for leishmania survival in the host (Ranjan and Dubey, 2024). The resistance of leishmaniasis to current treatments has driven researchers to explore different ligands. For example, a study on *L. major* modeled and simulated the GPI 14 molecule, finding that derivatives of N-4(-5(trifluoromethyl)-1-methyl-1H-benzo[d]imida-zole-2-yl)phenyl) could serve as anti-leishmanial drugs (Shinde et al., 2014). Additionally, computational methods have been employed to identify phytochemicals and known inhibitors for *L. donovani* adenosylmethionine decarboxylase through MDS and binding free energy calculations using the MM-PBSA method, identifying molecules such as Fagopyrine, Karpain, and Anabsinthin as promising candidates (Praffulla Kumar Arya et al., 2023). Furthermore, a multi-target approach using the PubChem database identified 203 compounds with potential anti-leishmanial properties, of which 15 ligands showed promise, with one ligand emerging as a particularly strong inhibitor (Saha and Nath Jha, 2023). An MDS study of *L. major* GP63 protein in water was also conducted. The results, using the GROMACS simulation program, found that the 1LML coded GP63 protein structure obtained from the Protein Data Bank was compatible with the substrate recognition and (pro)enzyme activation played by the N-terminal domain of GP63. A systematic analysis among a series of 10 homologues of GP63 showed that the amino acids involved in the interdomain bend are highly conserved (Bianchini et al., 2006). In a study conducted on screen inhibitors against *L. donovani*, potent natural compounds were investigated by applying cytotoxicity tests. Quercetin-3-rutinoside (Rutin) gave the best docking result from a library of 5,000 natural compounds, and a 100-ns MDS showed that Rutin could be a strong inhibitor (Kant et al., 2022). Studies have used MDS to investigate the inhibition of the mitochondrial enzyme, which is considered a drug target against *L. donovani* with commercially known ligands, performing active site determinations, molecular docking, and interaction analyses, and 300-ns simulations using MDS programs such as Desmond (Nath et al., 2024). Vaccine development studies are being carried out for CL caused by *L. major*, which is an important public health issue. In one of these studies, considering the GP63 glycoprotein, the 3D structure of the vaccine was developed, and a 100-ns simulation was performed using molecular docking and the Amber 20 MDS program to observe that the vaccine was correctly bound to the relevant structure (Naz et al., 2023). The ineffectiveness and development of resistance of conventional antileishmanial drugs against *Leishmania* species underscore the need for new therapeutic options (Zhang et al., 2025). It has been reported that there are two ways to develop new drug therapies; one is to find new drugs and the other is to optimize certain drug formulations (Dasauni et al., 2021). Therefore, the identification of parasite-specific proteins has become an urgent priority for the development of novel antileishmanial drug targets. Protein data obtained from structural protein databases have been reported to be highly valuable for structure-based ligand design. Moreover, MDS studies are frequently employed alongside molecular docking to determine accurate binding modes, binding energies, and solvation effects (De Vivo et al., 2016).

In light of these data, this study aimed to generate accurate structural models of key *L. tropica* ITS1 gene proteins through MDS and identify a novel ligand, benzo[d][1,3]dioxol-5-yl 4-acetamidobenzenesulfonate, that might act as an inhibitor with high therapeutic potential.

This study is expected to make a significant contribution to an anti-leishmanial drug design.

## 2 Materials and methods

### 2.1 Structure selection and preparation

**Figure 1:**
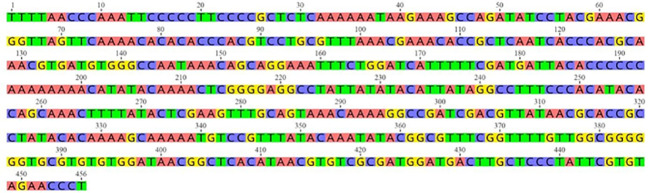
Nucleotide sequence of *Leishmania tropica* sample MH347948.1 (https://www.ncbi.nlm.nih.gov/nuccore/MH347948.1?report=genbank).

The FASTA nucleotide sequence of the ITS1 gene region of *L. tropica* was retrieved from the GenBank under the accession number MH347948.1 (Yentur Doni and Gurses, 2018; Doni et al., 2020) (https://www.ncbi.nlm.nih.gov/nuccore/MH347948.1) (**Figure 1**). This FASTA sequence was previously characterized through molecular typing and DNA sequence analysis to confirm its identity and genetic composition. For further analysis, the nucleotide sequence was translated into a protein sequence using the Expasy Online Translation Tool, which is available at https://web.expasy.org/translate/ (SIB). *L.tropica*, one of the *Leishmania* derivatives whose DNA sequence was previously determined from a bloodless, serous fluid sample taken from a patient with CL, was targeted (Doni et al., 2020). First, the protein structure of the sample was predicted based on the FASTA DNA sequences of the *L. tropica* ITS1 gene region. After determining the protein structure, benzo[d][1,3]dioxol-5-yl 4-acetamidobenzenesulfonate, a novel ligand not available in the literature, was docked to the sample using molecular docking. A 300-ns simulation was performed using the best score value. Analysis operations were carried out using the trajectory files obtained from the simulation. The results indicated that the ligand applied to *L. tropica* affected the protein structure significantly.

### 2.2 Preparation of the protein structure

**Figure 2:**
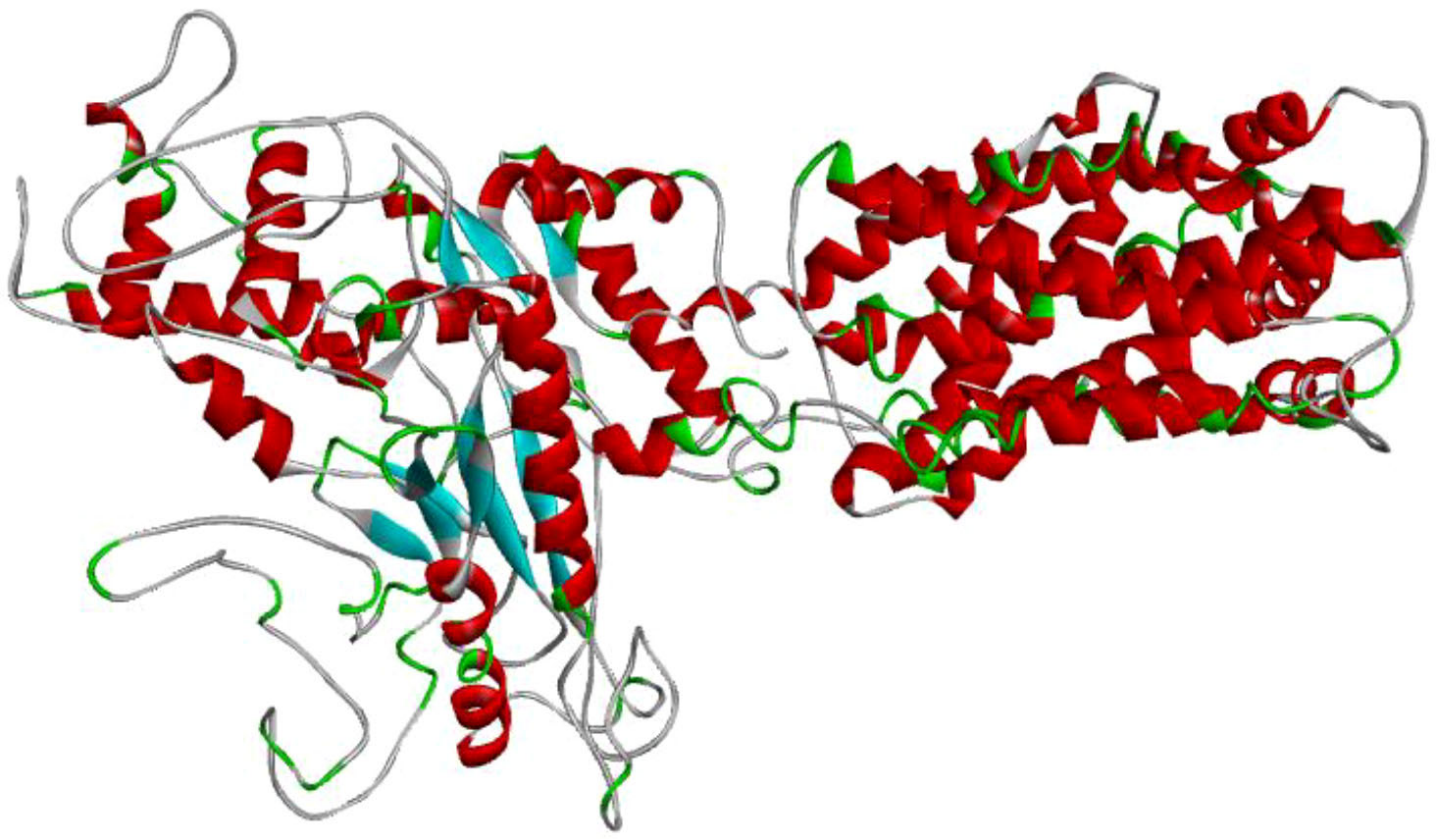
Protein structure of the sample *L. tropica* obtained using the I-TASSER modeling method.

Iterative Threading ASSEmbly Refinement (I-TASSER) was used to predict the protein structure of the sample from which the protein sequence was obtained (https://zhanggroup.org/I-TASSER/). Among the 5 protein structure prediction results, the structure with the best C-score range [−5,2] and the highest cluster density value was preferred (Yang and Zhang, 2015; Zheng et al., 2021; Zhou et al., 2022) (**Figure 2**).

### 2.3 Ligand selection

**Figure 3:**
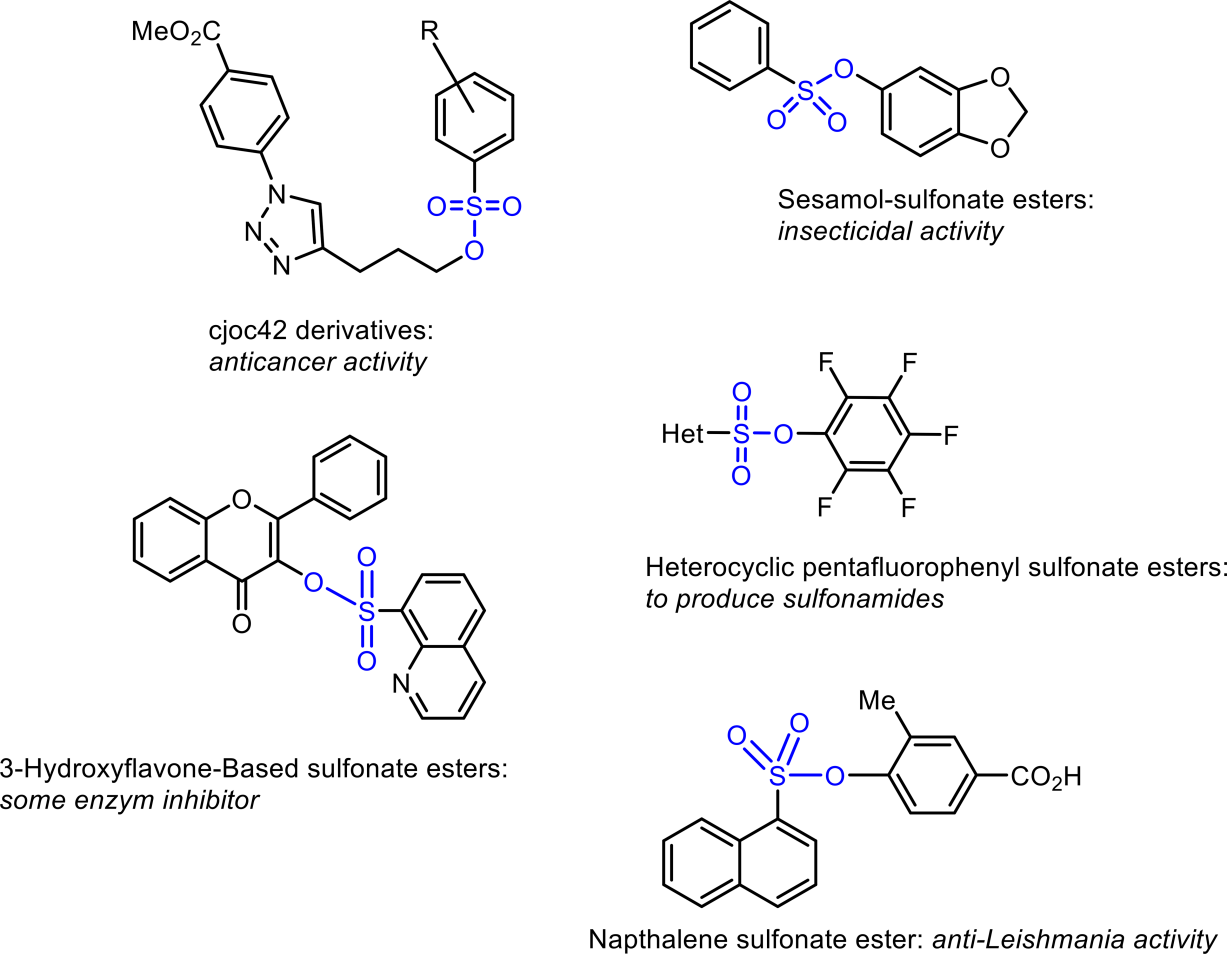
Biologically and synthetically important sulfonate esters.

Sulfonate esters (**Figure 3**), in addition to being important both synthetically and biologically, also appear as important key molecules in the synthesis of many molecules. For example, Che and his group synthesized many sulfonate esters with insecticidal properties based on sesamol (Che et al., 2020). Also, Kanabar and his group synthesized cjoc42-based sulfonate esters and showed that they had antiproliferative activity against pediatric liver cancer cell lines Hep3B and HepG2 (Kanabar et al., 2020). Researchers were closely interested in the synthesis of sulfonate esters, and many methods have been introduced to the literature (Luu et al., 2022). In addition, sulfonate esters have also been used as intermediate compounds in the preparation of molecules with important biological activity. Sulfonamides, known as synthetic antibiotics, were successfully synthesized from sulfonate esters by Bornholdt et al. (2009). Some enzymes that are targets in the treatment of diseases were also inhibited by molecules in the sulfonate ester structure (Guney et al., 2023).

**Figure 4:**
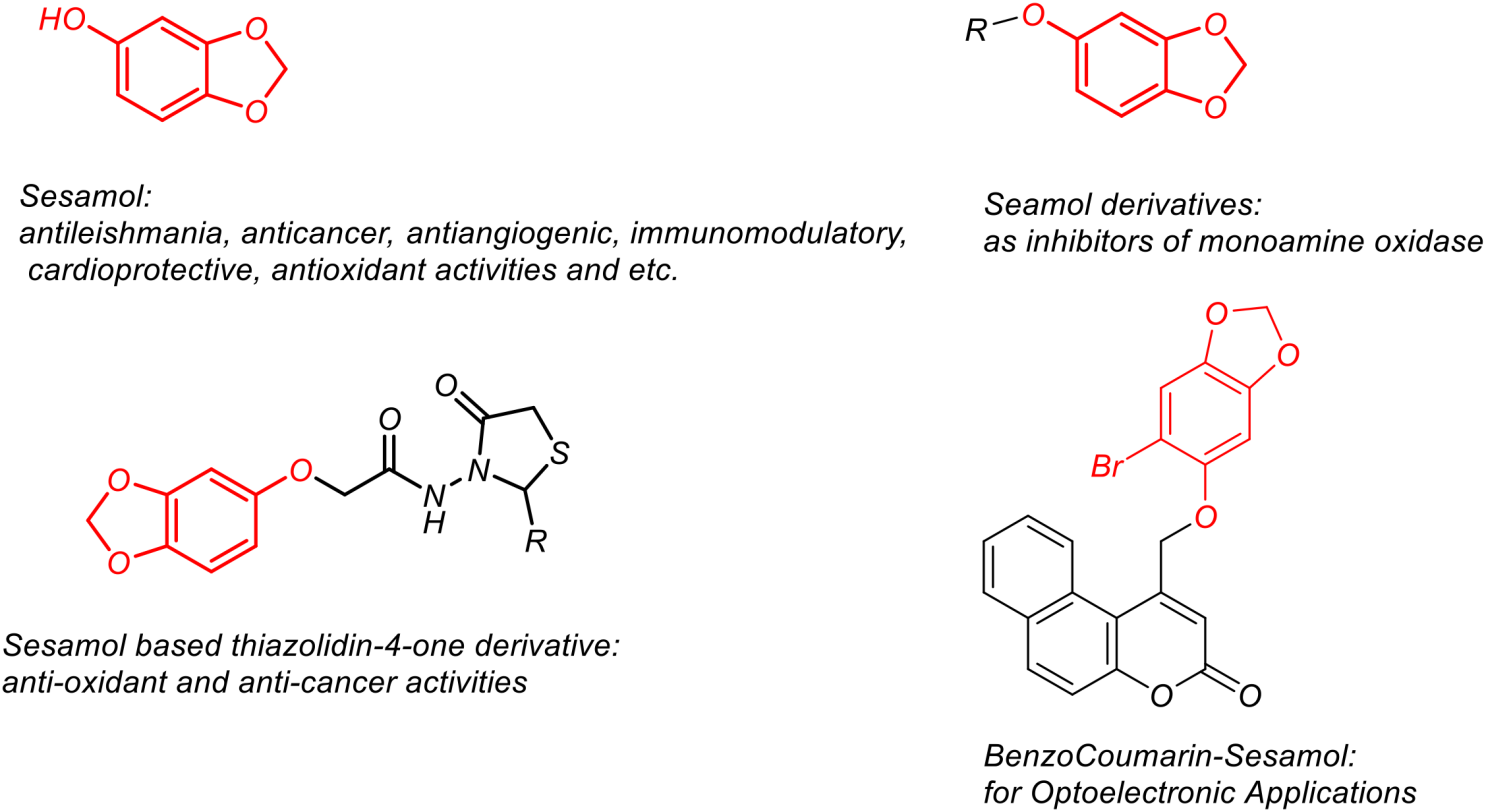
Sesamol and derivatives.

Sesamol (1), a naturally occurring phenolic compound, and its derivatives (**Figure 4**) have a wide range of biological activities (Engelbrecht et al., 2015; Yaswanatha Kumar et al., 2021; Kumar et al., 2024; Li et al., 2024). In addition, sesamol derivatives have attracted the attention of researchers with their optical properties (Madar et al., 2024). Studies on the pharmacokinetic properties and biodistribution of sesamol show that this compound can be distributed to organs, thus presenting a promising situation in terms of drug development for diseases (Khan et al., 2016). Although sesamol is a molecule that satisfies Lipinski rules (Geetha et al., 2015), the low bioavailability of sesamol, its rapid excretion, and toxic effects may be obstacles on clinical investigations (Singh et al., 2023). To overcome this obstacle, drug delivery system-based formulations of sesamol are being developed (Nair et al., 2023). In addition, substituted sesamol with groups such as alkyl and sulfonyl groups also allows for the synthesis of biologically important molecules (Palheta et al., 2020; Che et al., 2023). Sulfonate esters are also biologically important molecules. The negative interactions of sulfonate esters with DNA should also be kept in mind (Guney et al., 2023). In addition to all these biological activities, it has been shown that both sesamol and sulfonate esters may be beneficial in the treatment of leishmaniasis. For example, it has been stated that naphthalene sulfonate esters are potential inhibitors against leishmaniasis (Li et al., 2021), while sesamol is a plant-derived product that can be used against growth and cell proliferation in leishmaniasis (Ali et al., 2021). Based on the hypothesis that sesamol and sulfonate ester scaffolds may exert a synergistic effect against Leishmania, this study describes the synthesis of a sesamol-based sulfonate ester (ligand 3). Furthermore, molecular docking studies were conducted against proteins associated with the L. tropica ITS1 gene region to provide a framework for future anti-leishmanial research. Furthermore, research into the synthesis of new molecules with antileishmanial effect remains current, driven by concerns about resistance, cost, and toxicity (Avendano Leon et al., 2024; Chhajer et al., 2024). Based on all the effects and results obtained, it is believed that the synthesized compound 3 will contribute to the synthesis of new therapeutic compounds and drug development studies.

**Figure 5:**
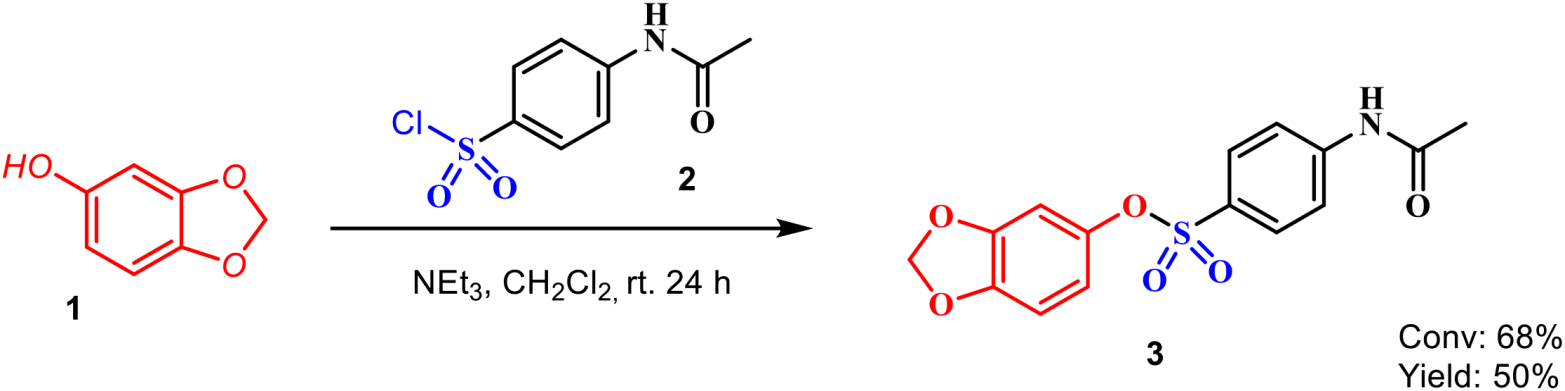
Synthesis of sulfonate ester 3.

For this purpose, sesamol-based sulfonate ester 3 was obtained by the traditionally used method. For this, sesamol (1) was converted into sulfonate ester 3 with 4-acetamidobenzenesulfonyl chloride in the presence of triethyl amine as the base and in CH_2_Cl_2_ as the solvent (**Figure 5**).

**Figure 6:**
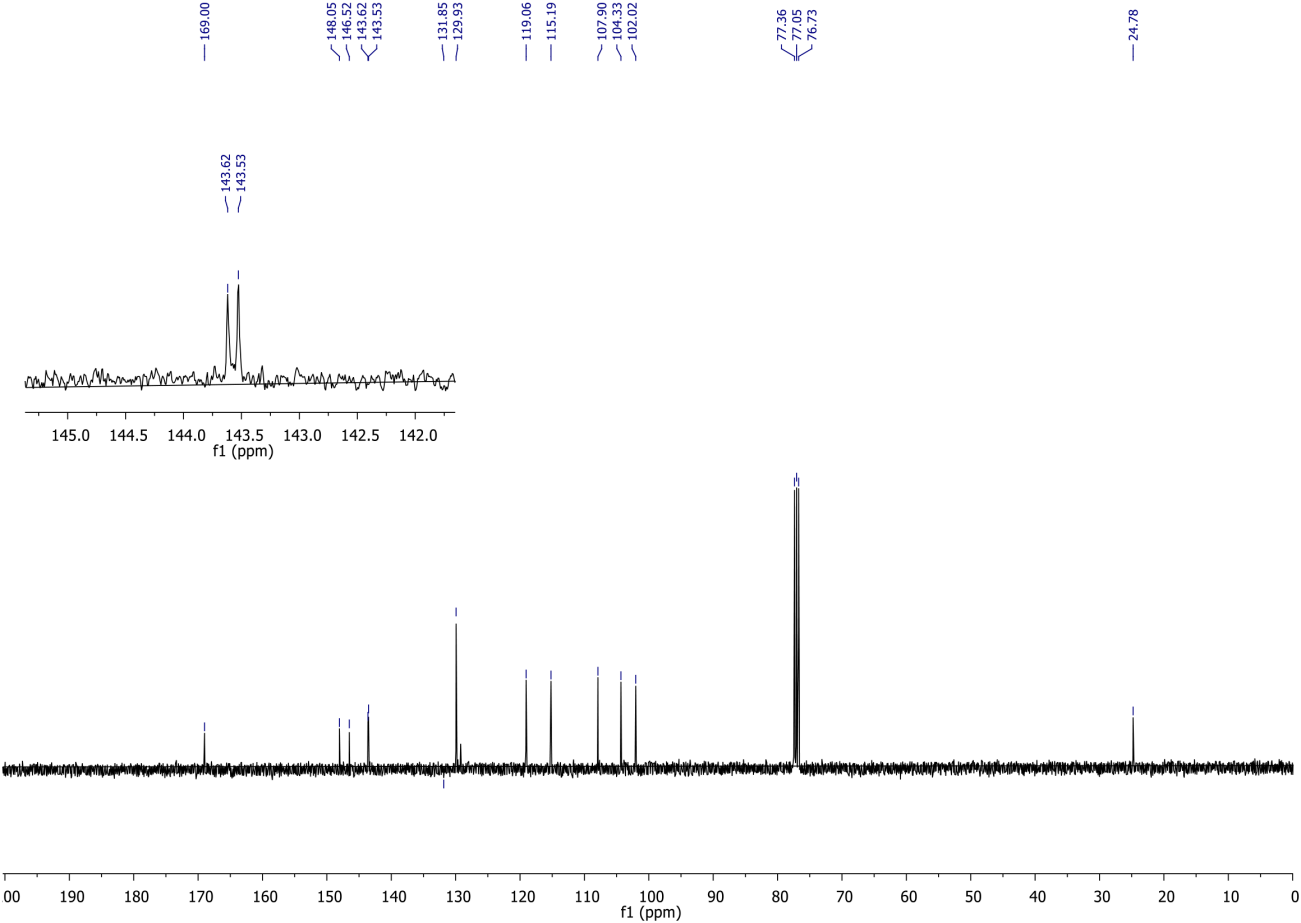
^13^C-NMR (101 MHz, CDCl_3_) spectra of compound 3.

The structure of the resulting compound was elucidated by NMR and IR spectra. When the ^1^H-NMR spectrum of the sulfonate ester 3 molecule is examined, the broad singlet at 7.89 parts per million (ppm) belongs to the amide NH proton. The protons in the aromatic ring to which the amide group is attached resonated as multiplet at 7.83 ppm. The peaks belonging to the sesamol ring resonated at 6.63, 6.52, and 6.37 ppm as doublet, doublet, and doublet of doublet, respectively. In the dioxol ring, CH_2_ protons resonated at 5.96 ppm as singlet. The singlet peak observed at 2.33 ppm belongs to the methyl protons in the amide group. The ^13^C-NMR spectrum (**Figure 6**) of sulfonate ester 3 is also in agreement with the structure. The carbon peaks of the aromatic ring to which the sulfonyl group in the molecule is attached and the peaks of the sesamol ring resonated between 102.46 and 148.08 ppm, with a total of 11 peaks. However, the amide carbonyl peak resonated at 168.95 ppm and the carbon belonging to the CH_3_ group attached to the carbonyl group resonated at 24.78 ppm.

### 2.4 Experimental

^1^H NMR and ^13^C NMR spectra were recorded on a Bruker (400 MHz) spectrometer. The IR spectra were determined using an FTIR spectrophotometer-Thermo Scientific Nicolet IS10.

#### 2.4.1 General procedure for the synthesis of sulfonate ester 3

**Figure 7:**
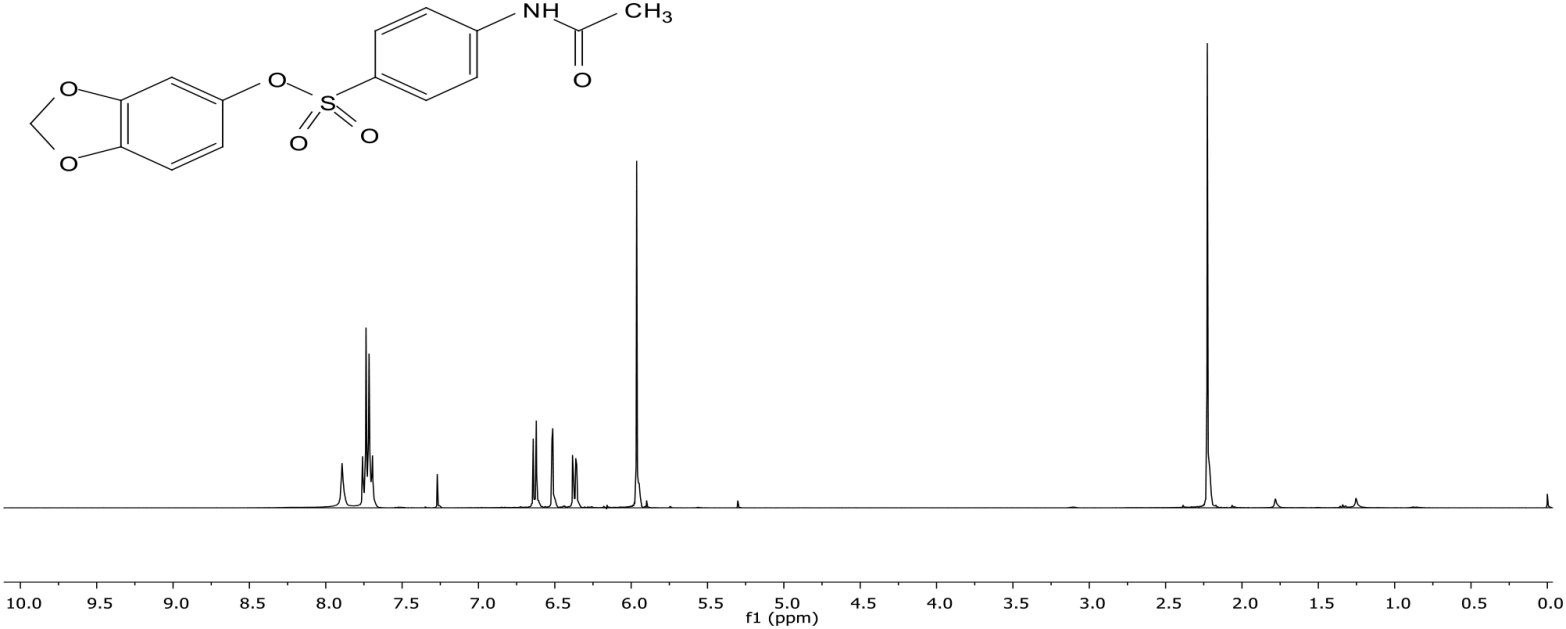
^1^H-NMR (400 MHz, CDCl_3_) spectra of compound 3.

**Figure 8:**
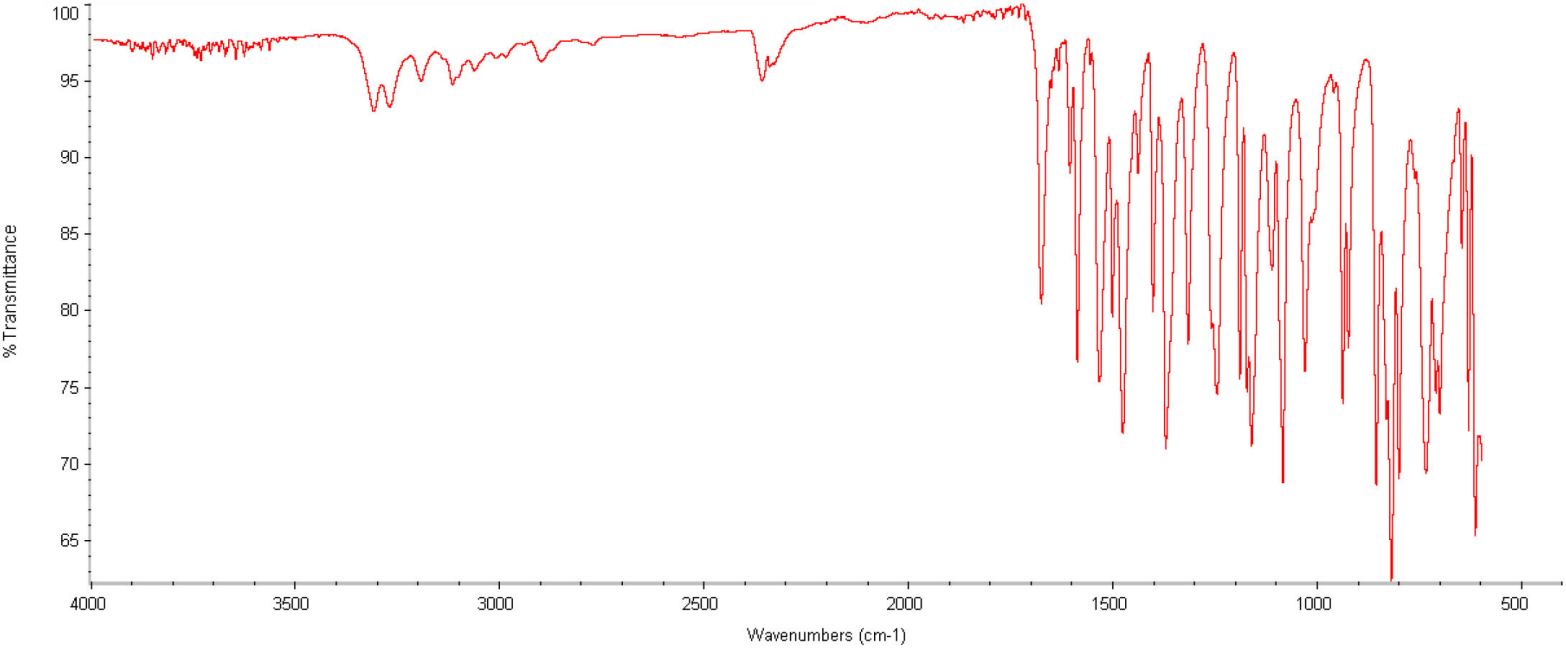
IR spectra of compound 3.

Sesamol (1) (7.24 mmol, 1.00 g) was taken into a 100-mL flask and dissolved in CH_2_Cl_2_ (20 mL) at room temperature, and then triethylamine (NEt_3_) (7.24 mmol) was added dropwise. After adding 4-acetamidobenzenesulfonyl chloride (2) (7.24 mmol), the resulting mixture was stirred at room temperature. After 24 h, the reaction mixture was diluted with water (15 mL) and extracted with DCM (30 mL × 3). Then, the combined organic phases were washed with saturated brine (30 mL) solution, dried over anhydrous Na_2_SO_4_, and filtered, and the solvent was removed *in vacuo*. After the removal of the solvent, in the ^1^H-NMR analysis (**Figure 7**), compound 1 was not obtained pure. All reactions of sesamol with 4-acetamidobenzenesulfonyl chloride (at low temperature and room temperature) gave the mixture of sulfonate ester 3 and the starting compound sesamol (1). After 24 h, sesamol (1) was converted to the product with 68%, and after recrystallization from methylene chloride/petroleum ether, sulfonate ester 3 was obtained with 50% yield (**Figure 8**).

Yield = 50%, white solid, (CH_2_Cl_2_/Petroleumether). m.p.: 139.5–140°C.

^1^H-NMR (400 MHz, CDCl_3_): δ = 7.89 (bs, NH), 7.83 (m, 4H), 6.63 (d, *J* = 8.5 Hz, 1H), 6.52 (d, *J* = 2.4 Hz, 1H), 6.37 (dd, *J* = 8.5 Hz, 2.4 Hz, 1H), 5.96 (s, 2H), 2.23 (s, 3H).

^13^C-NMR (101 MHz, CDCl3): δ = 169.00, 148.05, 146.52, 143.62, 143.53, 129.93, 129.21, 119.06, 115.19, 107.90, 104.33, 102.02, 24.78.

IR (ATR, cm-1 ν 3311, 3273, 3118, 1678, 1590, 1537, 1479, 1405, 1374, 1319, 1247, 1193, 1164, 1087, 1034, 941, 927, 859, 834, 822, 803.

### 2.5 Docking

In this study, a molecular docking study was carried out for the *L. tropica* ITS1 protein structure. The modeled molecule went through a preparation phase before the molecular docking study. The 2D structure of the ligand used for *L. tropica* was drawn with the Chem Draw program and optimized by selecting the MMFF9 force field with the Avagadro 1.2 program previously used in a study (Yorulmaz et al., 2022). As a result of this optimization, the relevant structure was saved in pdb format. During the docking study, only polar hydrogens and Kollman charges were added to the protein structure. Since a specific active site of the protein is not known in this study, the binding pose was investigated over the entire protein by blind docking. Accordingly, the selected grid parameters are given in **Table 1**.

**Table 1:** Grid parameters.

Grid box	Spacing (Å)	Grid center
126 126 78	1.0	94.122 94.309 96.377

Autodock 4.2.6 program was used for the molecular docking study (Ozturk et al., 2021). The simulations were carried out on a desktop computer with a Microsoft Windows 10 Education operating system, equipped with an Intel Core i5–2400 CPU 3.10 GHz dual processor and 4 GB of RAM.

**Figure 9:**
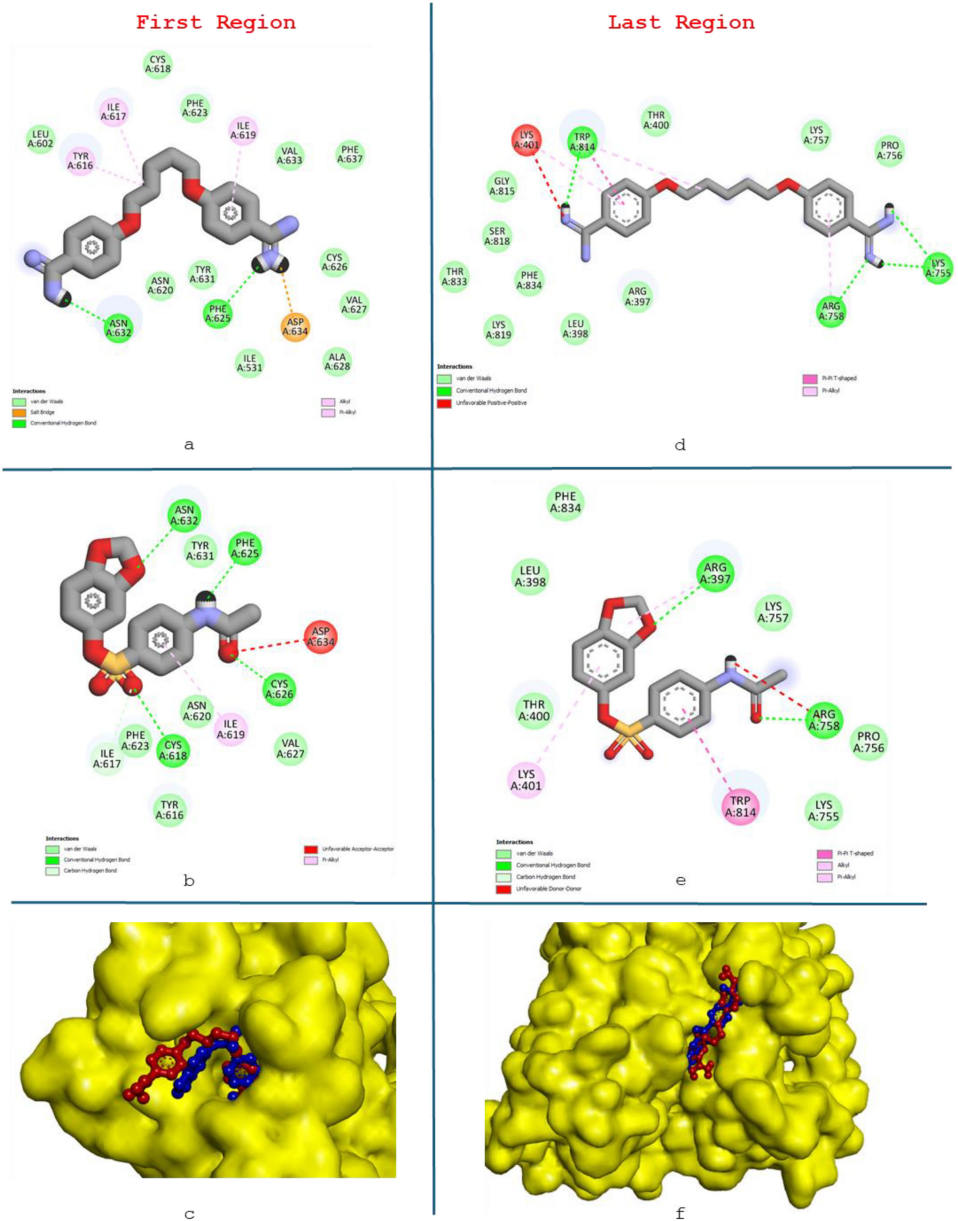
Ligand–protein interactions. **(a)** Pentamidine (first region), **(b)** benzo[d][1,3]dioxol-5-yl 4-acetamidobenzene-sulfonate (first region), **(c)** region where both ligands bind (first region), **(d)** pentamidine (last region), **(e)** benzo[d][1,3]dioxol-5-yl 4-acetamidobenzene-sulfonate (last region), **(f)** region where both ligands bind (last region).

In all docking simulations, the protein structure was chosen as rigid and the ligand was chosen as flexible. Each docking simulation was calculated using the Lamarckian genetic algorithm for 100 different ligand conformations at the binding site. In this study, pentamidine was selected as the reference ligand (de Boer et al., 2010; Javid et al., 2025). The pentamidine structure was obtained from the PubChem library in.sdf format. The optimized structure was converted to.pdb format and prepared for the docking study. Because of the novelty of the protein structure, blind docking was performed to determine the active site. Both pentamidine and the ligand studied in this study bound with the highest binding energy in the same region. This indicates that the region is functionally favorable for the protein and a pocket amenable to ligand binding. The blind docking grid parameters for the MDS starting position (0–140 ns) are as follows: Number of points (126 126 78), Center Grid Box (94.112 94.309 96.377), and Spacing 1.00 Å. Defining the grid parameters in the same way for both ligands makes the comparison methodologically consistent. In the MDS, the benzo[d][1,3]dioxol-5-yl 4-acetamidobenzenesulfonate ligand attached to a second active site in the 140–300 ns range. Docking was performed at this binding site for both pentamidine and benzo[d][1,3]dioxol-5-yl 4-acetamidobenzenesulfonate, and binding energies were compared. The relevant binding site was determined by selecting the last frame from the MDS file and identifying the surrounding amino acids. The grid parameters of the docking study performed at the second binding site are as follows: Number of points (106 80 98), Center Grid Box (138.101 72.387 90.555), and Spacing 0.375 Å. **Table 2** shows the docking results. Similarly, **Figure 9** shows the pockets to which both molecules bind and the types of interactions by which they bind to the protein.

**Table 2:** Docking results of the best bonding pose.

Ligand name	Binding energy (kcal/mol)	Inhibition constant, *K*_i_	Hydrogen bonds	The distance of hydrogen bonding (Å)
First binding region				
Pentamidine	−4.02	1.14 mM	H–PHE625:O	2.44
			H–ASN632:OD1	2.20
Benzo[d][1,3]dioxol-5-yl 4-acetamido-benzenesulfonate	−6.29	24.71 µM	CYS618:HN–O	2.66
			CYS626:HG–O	1.96
			H–PHE625:O	1.80
			ASN632:HN–O	2.34
Last binding region				
Pentamidine	−5.19	156.79 µM	ARG758:HN–N	2.22
			H–LYS755:O	2.34
			H–LYS755:O	2.00
			H–TRP814:O	1.98
Benzo[d][1,3]dioxol-5-yl 4-acetamido-benzenesulfonate	−5.60	78.60 µM	ARG758:HN–O	1.98
			ARG397:HE–O	2.73

When **Table 2** and **Figure 9** are examined, the docking results clearly show that the ligand proposed in the study establishes stronger and more stable interactions with the protein compared to pentamidine. The fact that both ligands bind to the same pocket at the first binding point determined by blind docking confirms that this region is a functionally active pocket for the protein; it is noteworthy that the benzo[d][1,3]dioxol-5-yl 4-acetamidobenzenesulfonate ligand, shown in blue in **Figure 9**, forms more and shorter-range hydrogen bonds than pentamidine, shown in red. In particular, the interactions established with PHE625, ASN632, and CYS residues support the fact that the benzo[d][1,3]dioxol-5-yl 4-acetamidobenzenesulfonate ligand exhibits higher binding affinity, consistent with lower binding energy and *K*_i_ values. A similar trend was observed in the second binding region identified in the later stages of the MDS; **Figure 9** shows that the ligand studied in this research forms effective hydrogen bonds with critical amino acids such as ARG758 and ARG397, resulting in better docking in the pocket. These results, which visually reveal the binding pockets and interaction types per the numerical docking data, demonstrate that the benzo[d][1,3]dioxol-5-yl 4-acetamidobenzenesulfonate ligand has a more advantageous binding profile compared to pentamidine in both the first and second binding sites, and stands out as a potential drug candidate.

### 2.6 Molecular dynamics simulation

A total of 2 different structures, namely, protein–ligand complex structures and *L. tropica* ITS1 gene in the apo state without ligand binding, were used for MDSs. All structures were prepared using the Solution Builder package (Jo et al., 2008; Lee et al., 2016, 2020) of the CHARMM-GUI program.

For MDSs, all ligand–protein complex systems were placed in 148 × 148 × 148 Å simulation boxes; TIP3-type water molecules were added and neutralized with 0.15 M NaCl. In addition to proteins and ligands, each system contains approximately 6,438 water molecules, 276 sodium (Na^+^) and 363 chloride (Cl^−^) ions, and approximately 14,298 atoms. The Charmm36m force field was used for all structures, and all systems were subjected to MDSs using computer systems with RTX A4000 GPU graphics cards with the GROMACS 2022.1 version software package.

In all systems, the temperature was gradually raised from 0 to 310 K and stabilized for 10 ns by applying a Nose-Hoover Thermostat (NPT) with a temperature coupling constant of 1.0 ps in the NVT section followed by a quasi-isotropic Berendsen barostat using a constant pressure group (NPT), and the pressure was kept constant throughout the MDS runs using a quasi-isotropic Parrinello–Rahman barostat. For each system, 300-ns simulation runs were performed to observe the formational changes in the protein resulting from the protein–ligand complex formation using periodic boundary conditions and a time step of 2 fs. All bonds were analyzed and constrained using the LINCS algorithm. Root mean square deviation (RMSD), root mean square fluctuation (RMSF), and distance analyses of the obtained MD trajectory files were performed using the gmx-molecular dynamics simulation package, which is part of the GROMACS software package. Hydrogen bonds, salt bridges, and hydrophobic interactions were analyzed to determine ligand–protein interactions.

### 2.7 *In silico* ADME/Tox analysis

SwissADME (Daina et al., 2017) was used to evaluate the drug-likeness properties (Lipinski’s rules, log*P*, TPSA, and solubility parameters) of the synthesized benzo[d][1,3]dioxol-5-yl 4-acetamidobenzenesulfonate (3).

ADMETlab 3.0 (Xiong et al., 2021) was applied to analyze the compound’s absorption (HIA, Caco-2 permeability, and P-gp interaction), distribution (PPB, Vdss, and BBB penetration), metabolism (CYP450 inhibition profile), elimination (clearance and half-life), and key toxicity endpoints (hERG inhibition, hepatotoxicity, DILI, and mutagenicity).

DataWarrior 6.1.0 (Sander et al., 2015) was employed to calculate the physicochemical properties (cLog*P*, TPSA, number of hydrogen bond donors/acceptors, and rotatable bonds), drug-likeness, and structural toxicity alerts (mutagenicity, tumorigenicity, reproductive toxicity, and irritation) of the compound.

ProTox-3.0 (Banerjee et al., 2018) was used to predict the acute toxicity (LD_50_), organ toxicities, and potential toxicological pathways of the compound; the corresponding toxicity classes were interpreted based on the probability scores provided by the model.

## 3 Results and discussion

**Figure 10:**
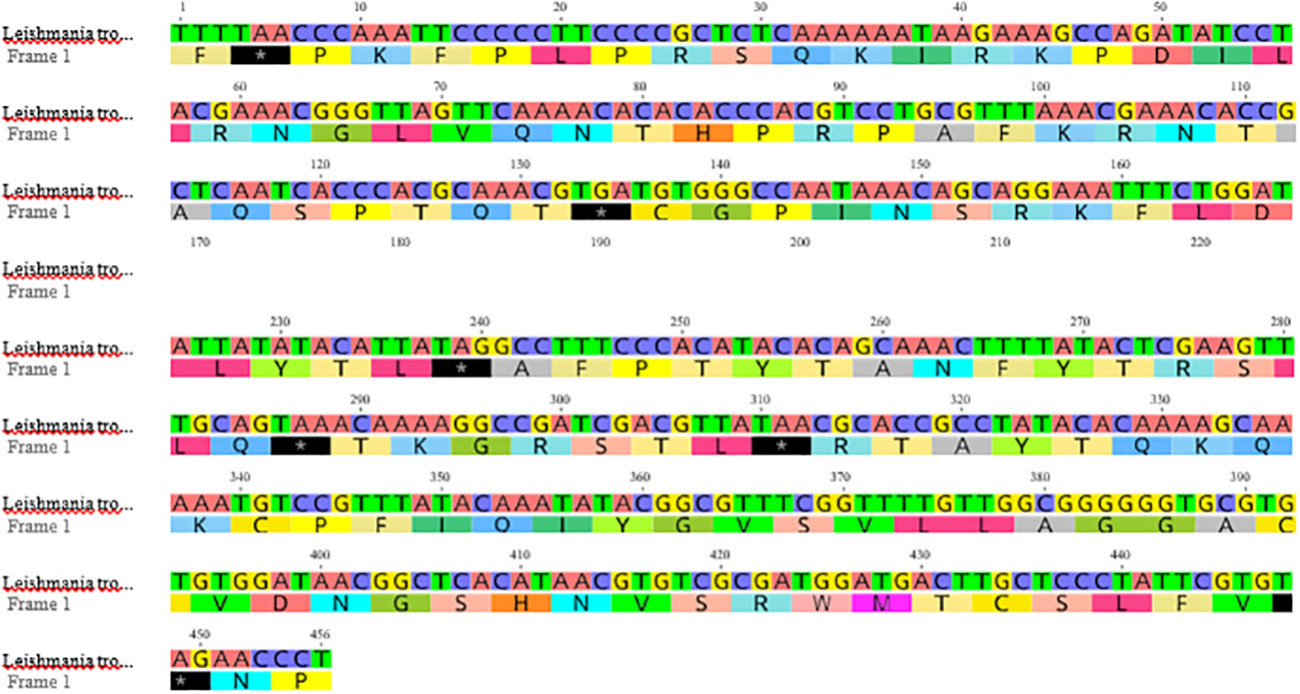
Sequence analysis and structural validation metrics for the predicted *Leishmania tropica* ITS1 gene protein (accession: MH347948) using I-TASSER.

To facilitate MDS analysis, the genomic sequences of Leishmania tropica were in silico translated into their corresponding amino acid sequences. This translation was performed using the ExPASy Translate tool provided by the Swiss Institute of Bioinformatics (SIB). **Figure 10** summarizes the quantitative validation of the 3D structures predicted via the I-TASSER platform, specifically detailing the C-score, TM-score, and RMSD values.

Using the protein sequence in **Figure 10**, the protein structure of the sample subject to the study was created via the I-TASSER online server (**Figure 2**). The I-TASSER modeling method yielded the five best model results (Zhang, 2008; Roy et al., 2010; Yang et al., 2015). Among the results, the model with the C-score value in the [−5,2] range and the highest cluster density value was preferred. The selected protein structure consists of a single chain and contains 14,262 atoms and 875 amino acids.

**Figure 11:**
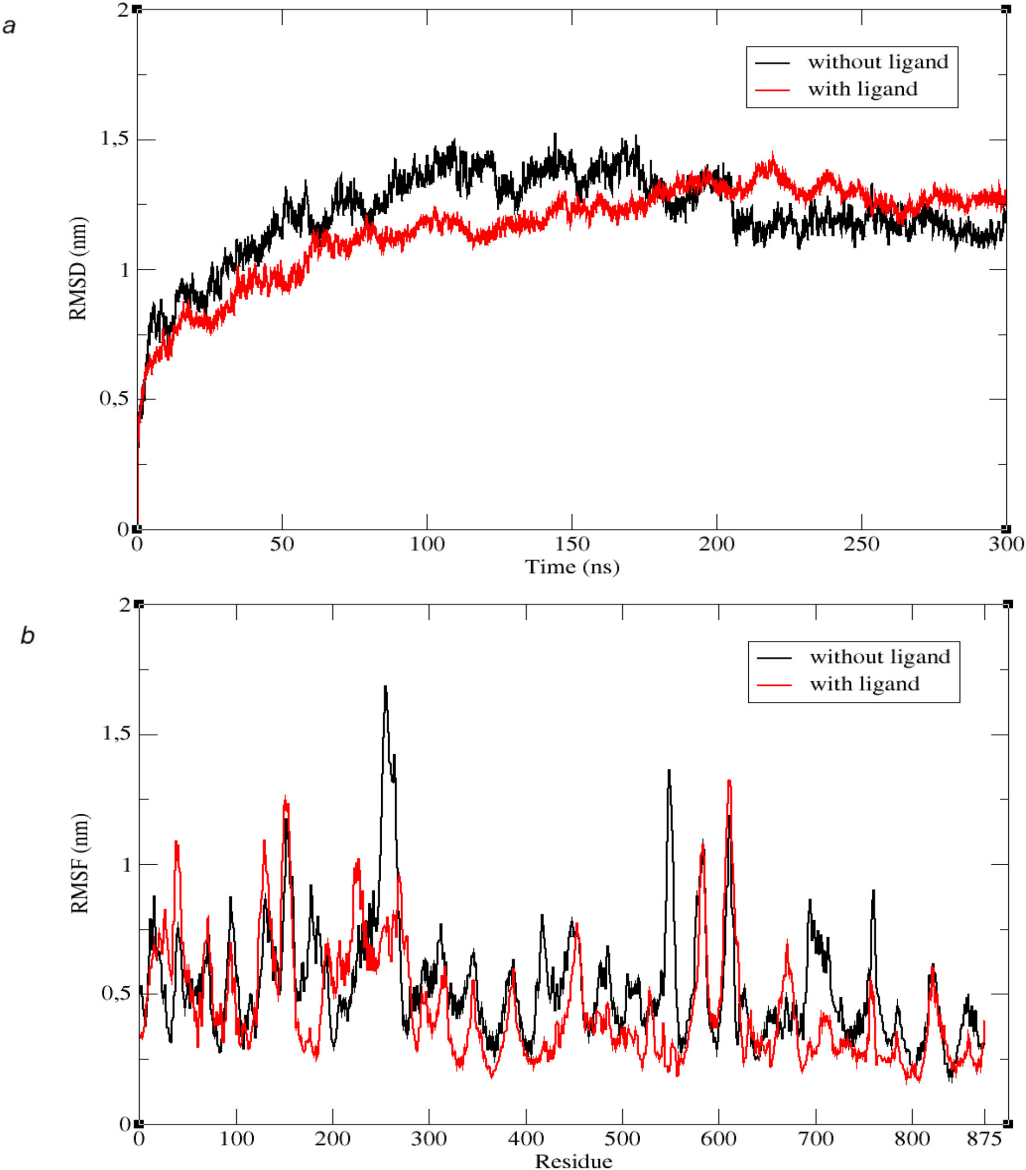
**(a)** RMSD values obtained as a result of the simulation for structures with ligand (red) and without ligand (black). **(b)** RMSF values obtained as a result of the simulation for structures with ligand (red) and without ligand (black).

The 300-ns simulation of the protein structure in **Figure 2** was performed with the GROMACS-2022 simulation program. When the RMSD graph obtained from the backbone of the structure as a result of the simulation is examined, it is seen that both the liganded and unliganded structures exhibit a steady state throughout the simulation (**Figure 11a**). This situation provides an idea that the simulation is performed properly. As a result of the simulation, the RMSF graph obtained for both with ligand and without ligand structures is as shown in **Figure 9b**. When the graph was examined, it was observed that most amino acids (70.29%) moved with lower fluctuation after the ligand was docked into the sample. This provides an idea that the relevant ligand may inhibit the *L. tropica* ITS1 gene region protein structure in general.

**Figure 12:**
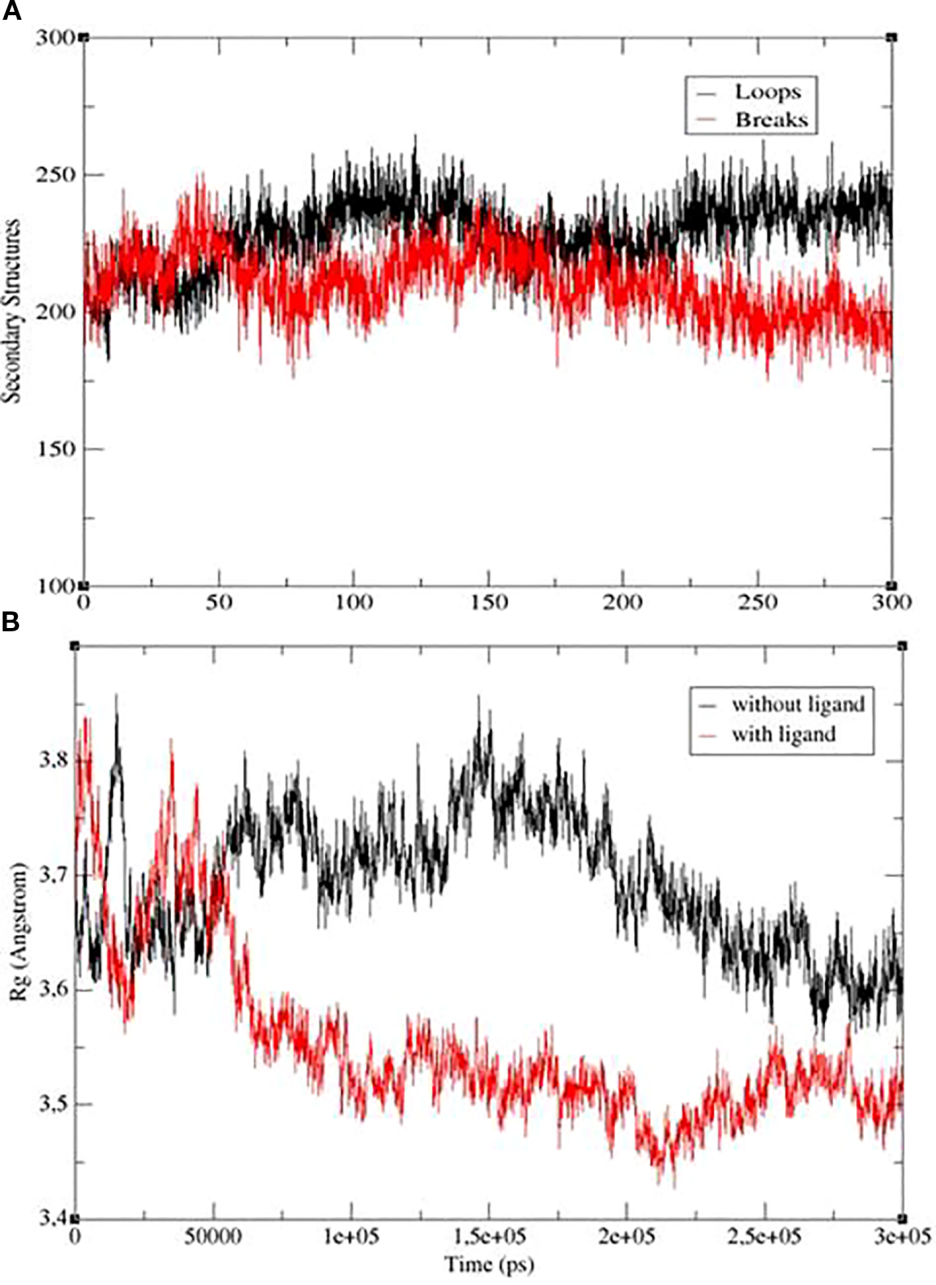
**(a)** Secondary structure values. **(b)** *R*_g_ values obtained as a result of the simulation for structures with ligand (red) and without ligand (black).

**Figure 12** shows the secondary structure and radius of gyration analyses. **Figure 12b** shows that the *R*_g_ values remained in the range of 3.5 to 3.8 Å for 300 ns, decreasing slightly towards the end of the simulation and stabilizing. The reason for the high RMSD values is that the protein is very large (875 residues), and the small fluctuations (0.2–0.3 Å) seen in the *R*_g_ graph cumulatively create a large RMSD in a massive structure. If there were any structural breakdown or unfolding, the *R*_g_ value would increase rapidly. The fact that *R*_g_ remained constant proves that the protein maintained its compact structure throughout the simulation and did not disintegrate like a tangle. Looking at the Dictionary of Protein Secondary Structure (DSSP) analysis shown in **Figure 12a**, it can be seen that the number of secondary structural elements (loops and breaks) is maintained throughout the entire trajectory. As a result, there is no significant transition from ordered structures (helices/sheets) to disordered states.

**Figure 13:**
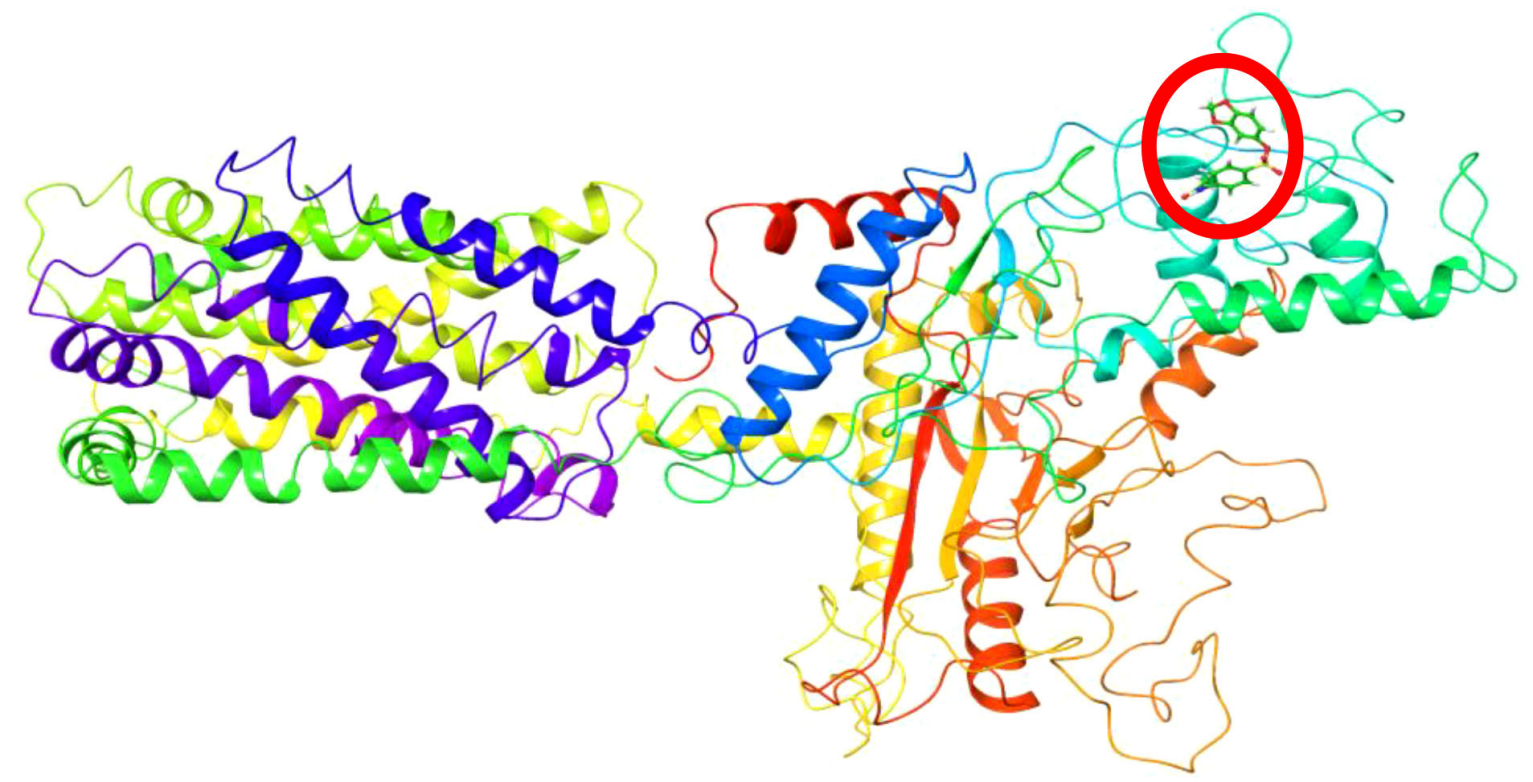
Protein structure obtained from the first frames.

**Figure 14:**
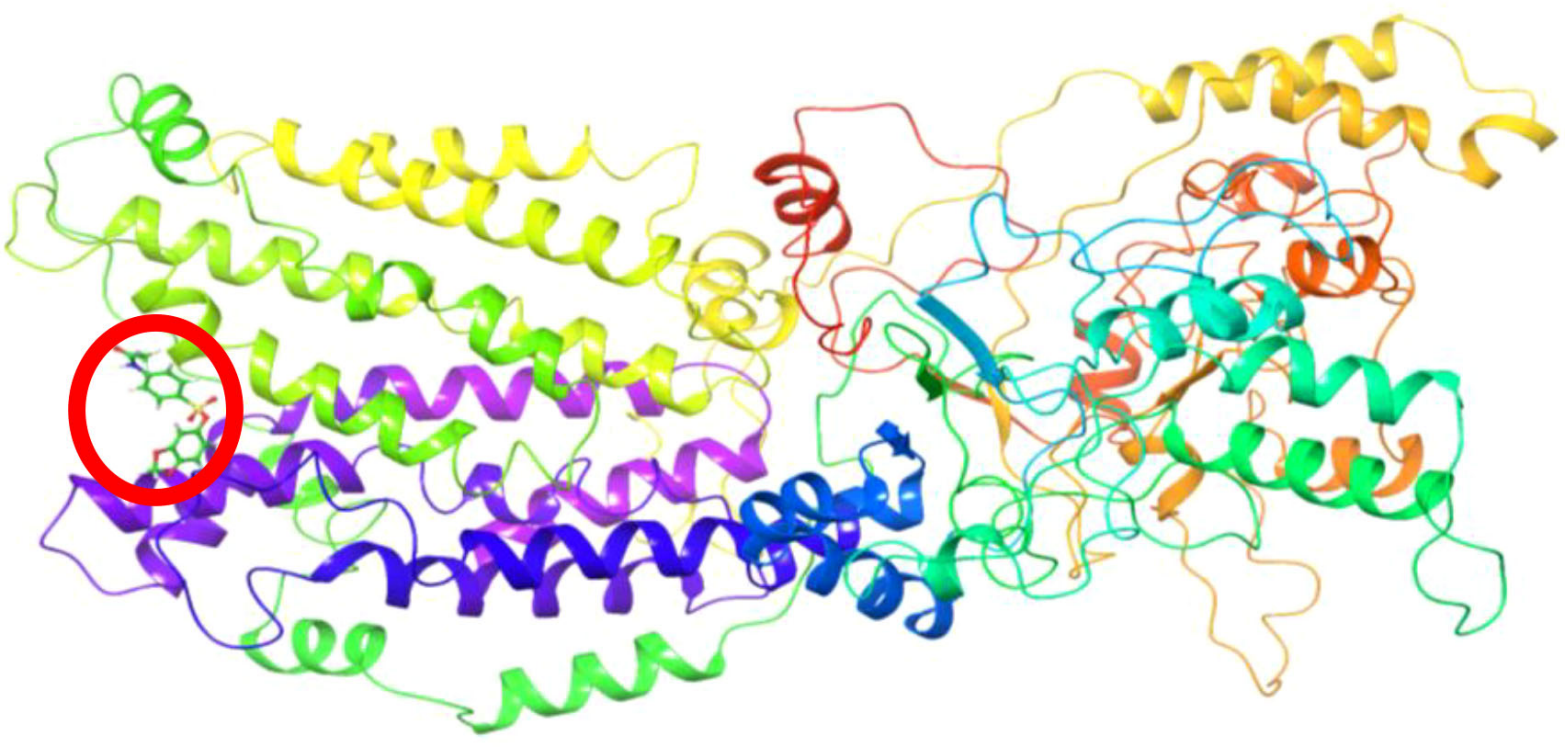
Protein structure obtained from the last frames.

**Figure 15:**
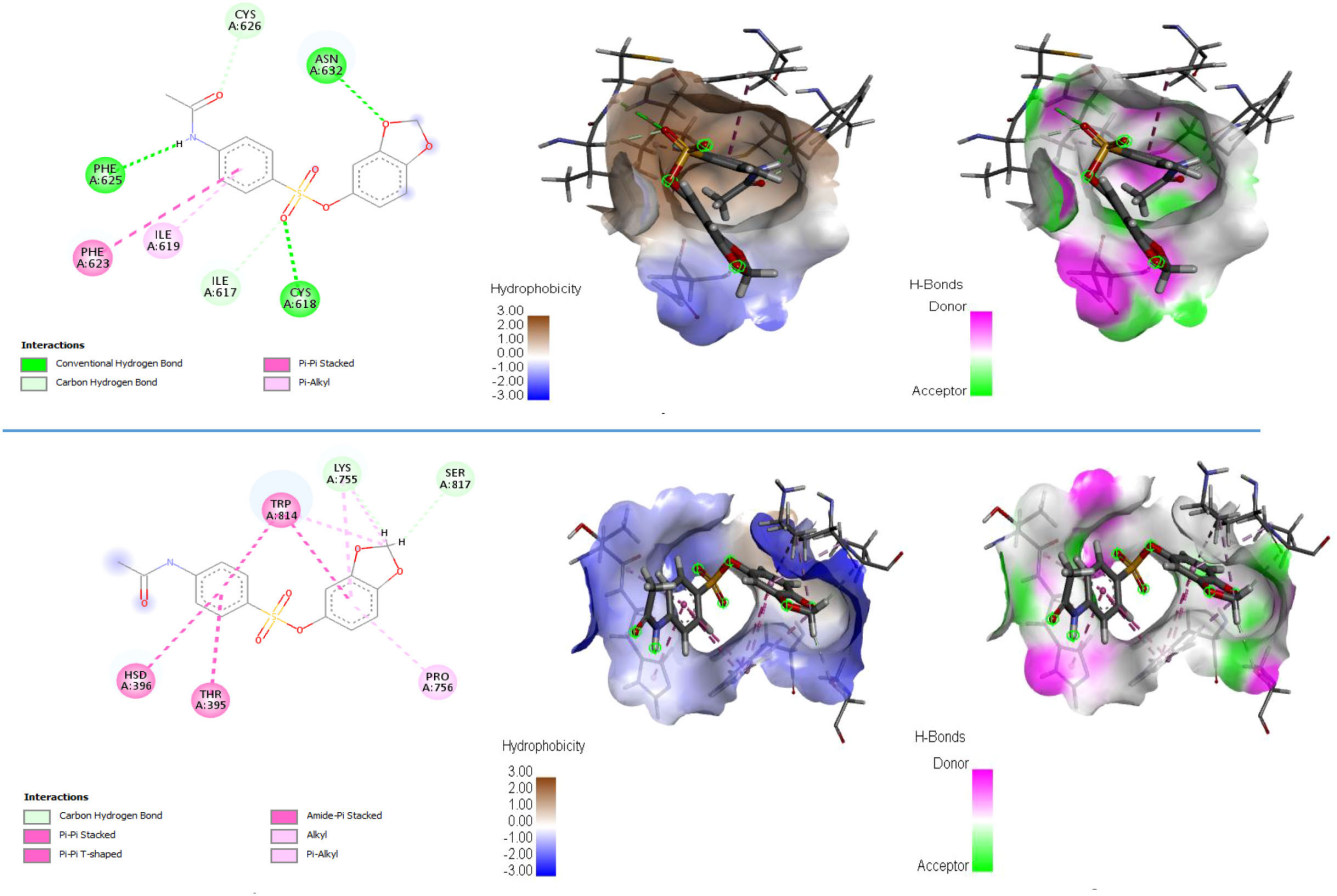
2D interaction graph **(a)**, hydrophobic **(b)** and H-bond interactions **(c)** of the protein structure obtained from the first frames. 2D interaction graph **(d)**, hydrophobic **(e)** and H-bonds interactions **(f)** of the protein structure obtained from the last frames.

In the analyses made using the trajectory files obtained as a result of the simulation, the ligand operates in the region specified in **Figure 13** for approximately the first 110 ns. It breaks away from the protein for approximately 25 ns and reconnects to the region indicated in **Figure 14**. The simulation remains committed to this region until the end. The hydrophobic interactions (b) and H-bond values (c) in the first bonded region are as shown in **Figure 15**. When the 2D interactions in the first position are examined, it was observed that it formed hydrogen bonds with ILE617, CYS618, PHE625, CYS626, and ASN632, and Pi interactions with ILE619 and PHE623 (**Figure 15a**). The hydrophobic interactions (e) and H-bond values (f) in the last bonded region are as shown in **Figure 15**. When the 2D interactions in the final position are examined, it was observed that it formed hydrogen bonds with LYS755 and SER817, and Pi interactions with THR395, HSD396, PRO756, and TRP814 (**Figure 15d**).

**Figure 16:**
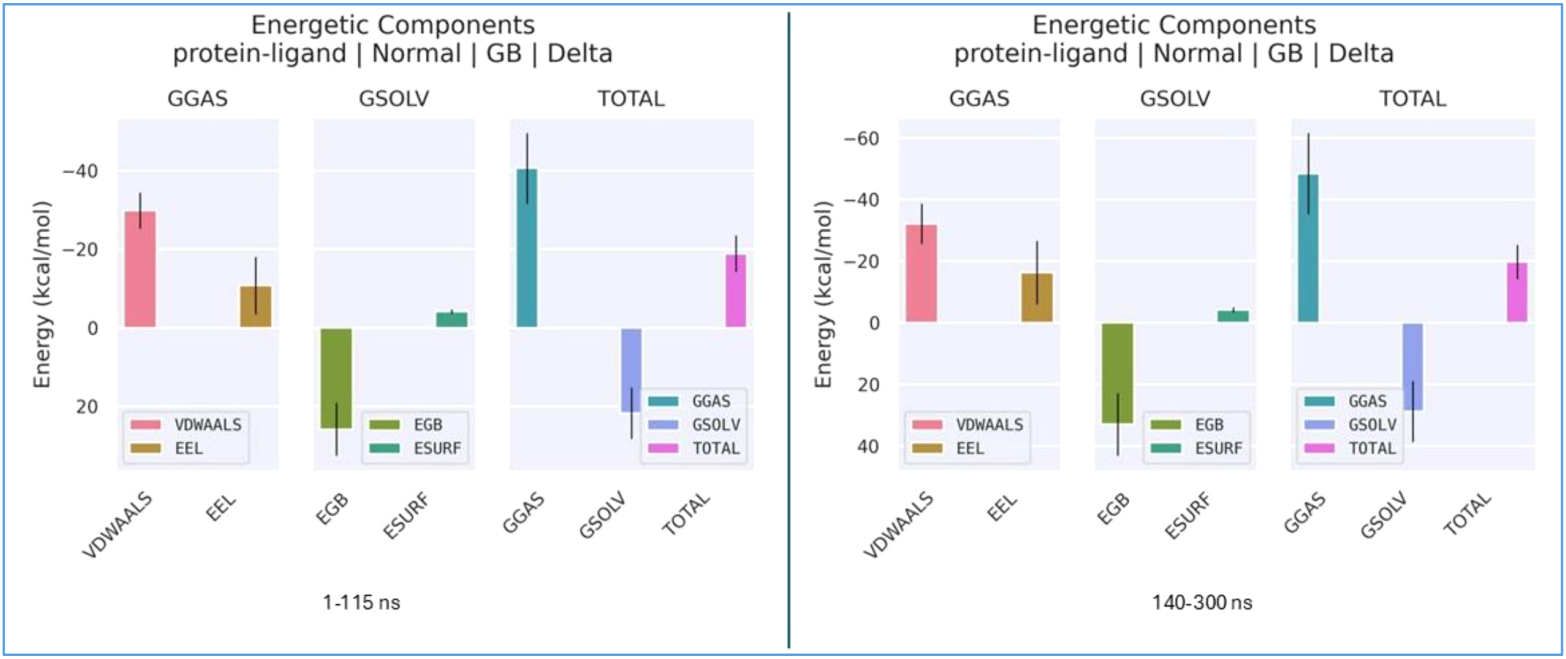
Total free energy plots for the two binding regions of the ligand.

To investigate the displacement of the ligand during the simulation, MM/GBSA and MM/PBSA analyses were performed. The total free energy plots for both states are presented in **Figure 16**. In both time intervals, the total free energy remained negative (ranging approximately from −18 to −20 kcal/mol). Notably, while the total energy was approximately −18 kcal/mol during the 1–115 ns interval, it decreased to approximately −20 kcal/mol between 140 and 300 ns. Following the 140-ns mark, the ligand transitioned to a second region with a more stable energy level, forming a thermodynamically superior complex. MM/GBSA analyses confirm that the ligand exhibits a higher affinity for the region occupied after 140 ns compared to the initial binding site. The average binding free energy improved from approximately −18.5 kcal/mol in the first region to −20.2 kcal/mol in the second, thereby enhancing the thermodynamic stability of the system. This increased stability was primarily attributed to the strengthening of van der Waals interactions (from −30 to −35 kcal/mol) and improved electrostatic complementarity.

**Figure 17:**
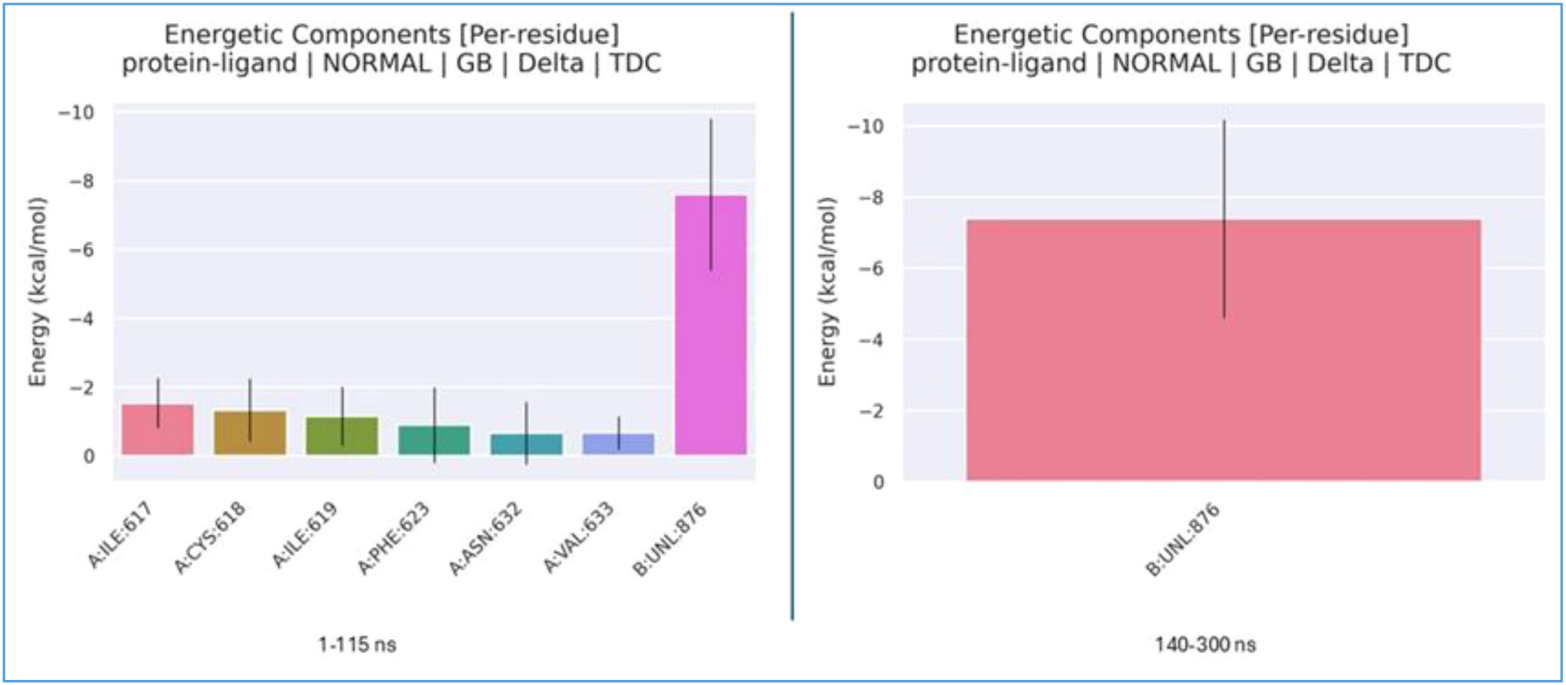
Per-residue energy decomposition plots for both binding regions of the ligand.

The per-residue energy decomposition values obtained from the MM/GBSA analysis are shown in **Figure 17**.

**Figure 18:**
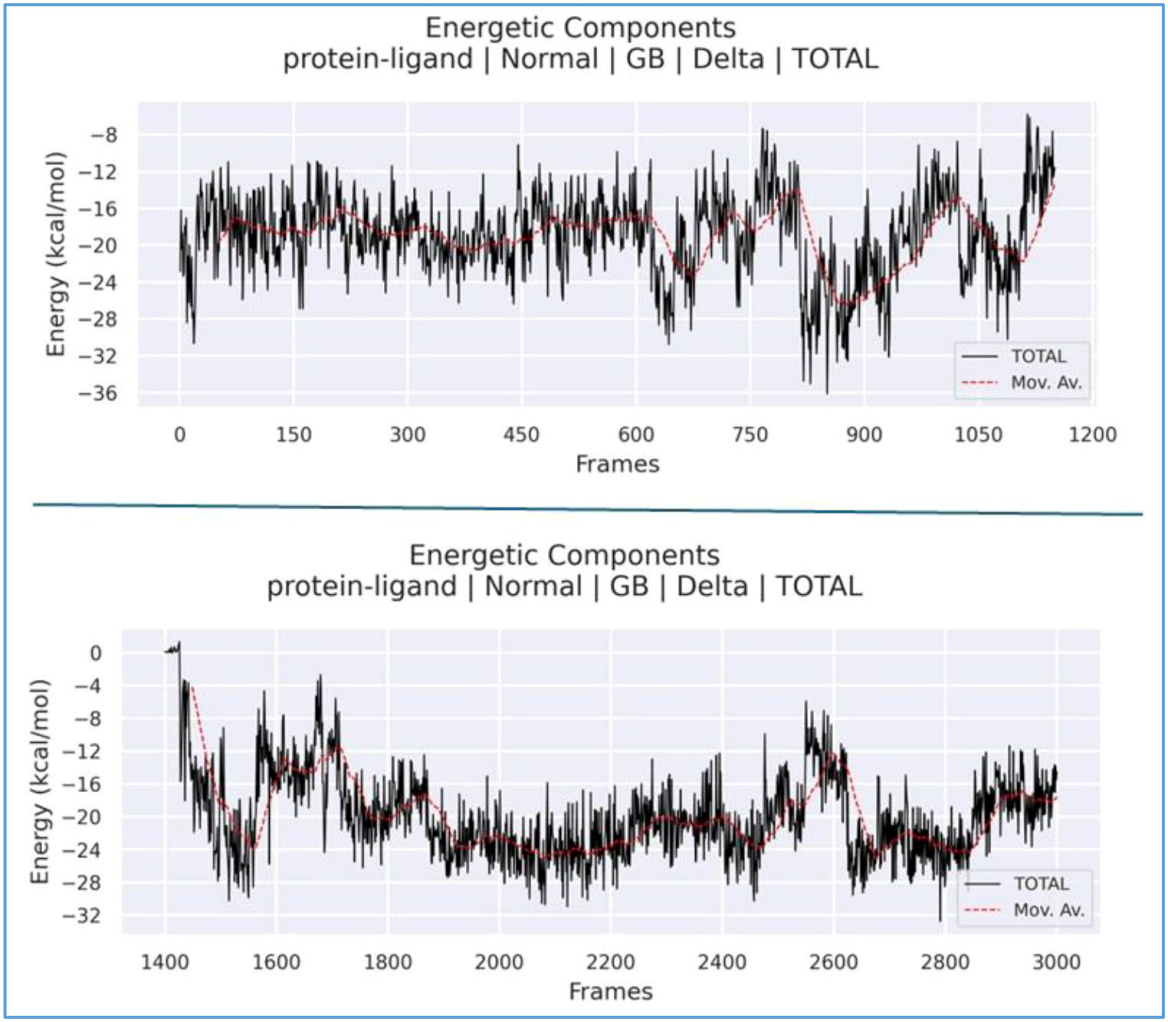
Total energy transition plots for both binding regions of the ligand.

Although *δ* remains negative in both regions, the binding energy during the 140–300 ns interval indicates greater stability compared to the initial region. In the second region, the van der Waals contribution decreased from approximately −30 to −35 kcal/mol. This indicates that the ligand achieves superior geometric complementarity with the protein surface in the second pocket, resulting in an increased contact surface area. Furthermore, while the electrostatic contribution in the first region (1–115 ns) was positive (repulsive), this repulsive force is markedly reduced in the second region. In the initial phase, the binding energy is distributed among several residues (e.g., ILE617, CYS618, ILE619, and PHE623), with each residue providing a relatively low contribution of approximately −2 kcal/mol. This distribution indicates a “loose” binding mode in that region. In contrast, the ligand exhibits a dominant energy contribution (approximately −7.5 kcal/mol) in the second region. While the ligand forms weak and diffuse interactions with various amino acids in the first region, it settles into a specific site within the protein after 140 ns, establishing significantly stronger and more specific interactions. This ligand displacement observed during the simulation is further supported by the MM/GBSA per-residue energy decomposition analysis. During the 1–115 ns interval, the ligand forms transient interactions with residues such as ILE617 and PHE623; however, from 140 ns onwards, it resides in its final, more stable pocket. In this new conformation, the increase in van der Waals interactions and the reduction in electrostatic repulsion allow the ligand to reach a lower free energy state within the protein structure. This quantitatively demonstrates that the second region represents the most stable binding mode for the ligand on the target protein. The corresponding energy transitions are illustrated in **Figure 18**.

In drug design, the *in silico* evaluation of the pharmacokinetic and safety profiles of candidate molecules is a critical step, and the assessments obtained for the synthesized ligand are presented in **Tables 3**–**6**.

**Table 3:** Physicochemical, pharmacokinetic, and drug-likeness parameters of the synthesized ligand predicted by SwissADME.

ADMET parameter	Prediction/value
MW (g/mol)	335.33
HBD	1
HBA	6
TPSA (Å²)	99.31
Log*P* (Consensus)	2.08
Log*S* (ESOL)	−3.24
GI	High
BBB	No
DL	Yes
P-gp substrate	No

**Table 4:** Pharmacokinetic and toxicity predictions of the synthesized ligand obtained from ADMETlab 3.0.

ADMET parameter	Prediction/value
Human intestinal absorption (HIA)	0.0
Caco-2 permeability	−4.90
P-gp substrate/inhibitor	−/−
Plasma protein binding (PPB, %)	96.6
Volume of distribution (Vdss, log L/kg)	−0.26
Blood–brain barrier penetration	0.602
CYP2C19 inhibition	+++
CYP2D6 inhibition	+++
CYP3A4 inhibition	+++
Clearance (CL)	0.99
Half-life (*T*½)	0.78
hERG inhibition	−−
Hepatotoxicity	+++

(–) low likelihood; (+) predicted positive interaction; (+++) strong likelihood; (– –) very low risk; (±) ambiguous model prediction; (–/–) no significant interaction.

**Table 5:** Physicochemical properties of the synthesized ligand predicted by DataWarrior.

DataWarrior	Prediction/value
Molecular weight	335.33
cLog*P*	2.35
Number of H-acceptors	7
Number of H-donors	1
Polar surface area (Å²)	99.31
Rotatable bonds	5

**Table 6:** Toxicity predictions of the synthesized ligand obtained from DataWarrior.

DataWarrior	Prediction/value
Mutagenic	None
Tumorigenic	None
Reproductive effect	None
Irritant	High

According to SwissADME analysis, the synthesized ligand complies with Lipinski’s rule of five and exhibits high gastrointestinal absorption. BBB penetration was predicted as negative, and the compound was not identified as a P-gp substrate. The consensus Log*P* value indicates moderate lipophilicity.

According to the ADMETlab 3.0 results presented in **Table 5**, the compound exhibits poor intestinal absorption (HIA = 0.0) despite moderate Caco-2 permeability. The high PPB value (96.6%) indicates extensive plasma protein binding. BBB penetration was predicted to be moderate (0.602), while the strong inhibitory profiles estimated for CYP2C19, CYP2D6, and CYP3A4 suggest a notable risk of metabolic interactions. In addition, the predicted very low hERG inhibition suggests a favorable cardiac safety profile, whereas the model-dependent variability observed in hepatotoxicity- and DILI-related predictions, together with the inherent limitations of *in silico* approaches, indicates that these results require experimental validation. These assessments are based on the statistical classification system implemented in ADMETlab (Xiong et al., 2021).

The compound showed no risk of mutagenic, tumorigenic, or reproductive toxicity; however, it exhibited a high irritation potential. Its physicochemical properties were consistent with Lipinski’s criteria, indicating an overall drug-like profile. According to the ProTox-3.0 analysis, the compound showed low acute toxicity (LD_50_ = 3,200 mg/kg; toxicity class V). A moderate risk was predicted for hepatotoxicity and nephrotoxicity, while no evidence of mutagenicity, immunotoxicity, or carcinogenicity was observed. In line with the very low hERG inhibition predicted by ADMETlab, no clear cardiotoxicity liability was identified; nevertheless, experimental studies are required to validate these *in silico* findings.

## 4 Conclusions

Consequently, I-TASSER is considered as a robust, peer-reviewed alternative for generating reliable tertiary models via threading and fragment assembly. The structural integrity of the resulting models was instead validated using I-TASSER’s native confidence metrics (C-score and TM-score). In this study, an *in silico* approach was employed to evaluate a novel ligand, benzo[d][1,3]dioxol-5-yl 4-acetamidobenzenesulfonate, as a potential inhibitor of the *L. tropica* ITS1 gene protein (875 amino acids). This research addresses the urgent need for novel antileishmanial drugs with enhanced efficacy and reduced side effects. During the 300-ns MDS, it was observed that the ligand remained bound to the first binding region for approximately 110 ns, dissociated for 25 ns, and then spontaneously bound to a second region where it remained stable for the final 165 ns. MM/GBSA trajectory analysis revealed a clear energetic preference for this second site, with binding energy stabilizing at approximately −22 kcal/mol after 140 ns. This stabilization primarily stems from increased van der Waals interactions, suggesting superior surface complementarity within the second pocket.

As the active site of the *Leishmania* ITS1 gene protein was previously unknown, this spontaneous migration strongly suggests that the second region constitutes a primary functional pocket. Our findings indicate that the ligand may not only inhibit the target protein but also serve as a tool for identifying active sites in novel structures. Furthermore, the ADMET profile demonstrated drug-like physicochemical properties and a lack of mutagenic or tumorigenic risks. However, predicted low intestinal absorption and high plasma protein binding suggest that further pharmacokinetic optimization is required. These results provide a strong foundation for future *in vitro* and *in vivo* biological activity evaluations to validate the candidate’s antileishmanial potential (Ranjan and Dubey, 2024).

## Data Availability

The datasets presented in this study can be found in online repositories. The names of the repository/repositories and accession number(s) can be found in the article/**Supplementary Material**.
