# Supplementary material for: Structural analysis of ITS 1 gene of Leishmania tropica and evaluation of a novel ligand, benzo[d][1,3]dioxol-5-yl 4-acetamidobenzenesulfonate, via molecular modeling methods
Source: Front Cell Infect Microbiol. 2026 Mar 3;16:1743630. doi: 10.3389/fcimb.2026.1743630 (PMC13040372; doi:10.3389/fcimb.2026.1743630)
Supplement: Supplementary file 1 [file DataSheet1.pdf]

Supplementary material

Toxicity Studies

In silico studies are used intensively by many researchers in drug design due to the advantages they provide(Shaker et al., 2021; Shin et al., 2023; Hashem et al., 2024). Toxicity effect also has an important place before clinical studies begin(Parasuraman, 2011; Sahin, 2022). One of the programs that allows the toxic effects of molecules to be examined in silico is Protox web server(Banerjee et al., 2024; Salem et al., 2024). The toxic evaluation of the synthesized sulfonate ester **3** was made according to Protox-III and the following results were obtained (https://tox.charite.de/protox3/index.php?site=compound_search_similarity).

i) The sulfonate ester **3** compound was found to have a low toxic value. (LD50: 3200mg/kg)

ii) Sulfonate ester **3** was found to be active in terms of dili, nephro, cardio, bbb, clinical, CYP2C9.

Table 1. Acute oral toxicity prediction obtained by using Protox-II web server.

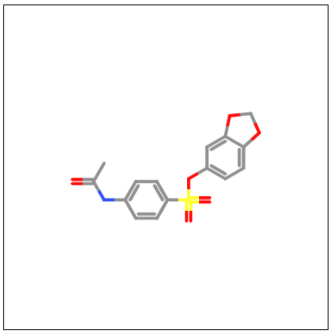

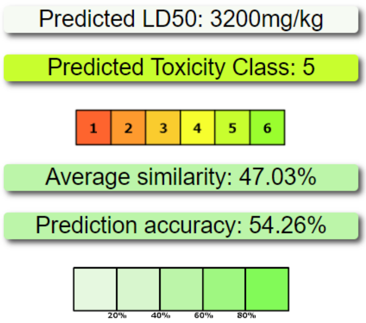

Name	CC(=O)NC1=CC=C(C=C (=O)OC1=CC=C2OCOC[illegible]
Molweight	335.33
Number of hydrogen bond acceptors	7
Number of hydrogen bond donors	1
Number of atoms	23
Number of bonds	25
Number of rotable bonds	5
Molecular refractivity	81.12
Topological Polar Surface Area	99.31
octanol/water partition coefficient(logP)	3.3

References

Banerjee, P., Kemmler, E., Dunkel, M., and Preissner, R. (2024). ProTox 3.0: a webserver for the prediction of toxicity of chemicals. *Nucleic Acids Res* 52(W1), W513–W520. doi: 10.1093/nar/gkae303.

Hashem, H.E., Ahmad, S., Kumer, A., and Bakri, Y.E. (2024). In silico and in vitro prediction of new synthesized N-heterocyclic compounds as anti-SARS-CoV-2. *Sci Rep* 14(1), 1152. doi: 10.1038/s41598-024-51443-7.

Parasuraman, S. (2011). Toxicological screening. *J Pharmacol Pharmacother* 2(2), 74–79. doi: 10.4103/0976-500X.81895.

Sahin, S. (2022). A single-molecule with multiple investigations: Synthesis, characterization, computational methods, inhibitory activity against Alzheimer's disease, toxicity, and ADME studies. *Comput Biol Med* 146**,** 105514. doi: 10.1016/j.compbiomed.2022.105514.

Salem, M.E., Abdelhamid, I.A., Elwahy, A.H.M., Ragheb, M.A., Alqahtani, A.S., Zaki, M.E.A., et al. (2024). Novel hybrid thiazoles, bis-thiazoles linked to azo-sulfamethoxazole: Synthesis, docking, and antimicrobial activity. *Heliyon* 10(10)**,** e31082. doi: 10.1016/j.heliyon.2024.e31082.

Shaker, B., Ahmad, S., Lee, J., Jung, C., and Na, D. (2021). In silico methods and tools for drug discovery. *Comput Biol Med* 137**,** 104851. doi: 10.1016/j.compbiomed.2021.104851.

Shin, H.K., Huang, R., and Chen, M. (2023). In silico modeling-based new alternative methods to predict drug and herb-induced liver injury: A review. *Food Chem Toxicol* 179**,** 113948. doi: 10.1016/j.fct.2023.113948.
